# Repression of interferon regulatory factor-4 (IRF4) hyperactivation restricts murine lupus

**DOI:** 10.1038/s41392-023-01413-8

**Published:** 2023-05-22

**Authors:** Shijun He, Huihua Ding, Li Chen, Yiwei Shen, Yuting Liu, Fenghua Zhu, Xiaoqian Yang, Nan Shen, Zemin Lin, Jianping Zuo

**Affiliations:** 1 Innovation Research Institute of Traditional Chinese Medicine, Shanghai University of Traditional Chinese Medicine, Shanghai, China; 2 Laboratory of Immunopharmacology, State Key Laboratory of Drug Research, Shanghai Institute of Materia Medica, Chinese Academy of Sciences, Shanghai, China; 3 Department of Rheumatology, Ren Ji Hospital, School of Medicine, Shanghai Jiao Tong University, Shanghai, China

**Keywords:** Translational immunology, Rheumatic diseases

**Dear Editor**,

Hyperactivation of the type I interferon (IFN) signature has been observed in several systemic autoimmune conditions, and the type I IFN receptor antagonist *Saphnelo* (anifrolumab-fnia) has been approved in 2021 by the Food and Drug Administration (FDA) in the US. The launch of *Saphnelo* marked the only new treatment approved for systemic lupus erythematosus (SLE) in more than 10 years.

Albeit several studies have been focused on the role of IFN-responding genes (IRGs) and IFN signature in rheumatic conditions,^1^ the link between IRGs regulators and systemic autoimmune conditions is poorly characterized. Interferon regulatory factors (IRFs) are a family of transcription factors that modulate immune responses through IFN signaling pathway. Among the nine mammalian members (IRF1-IRF9), IRF4 expression is restricted in immune cells and is required for their proper maturation and differentiation. A previous study using blood samples from SLE patients demonstrated that the differential expression of IRF4 and IRGs can delineate gene expression signatures associated with clinical features and treatment outcomes.^2^ However, prior research has not been aimed at clarifying the association among focal IRF4 expression, target organ pathology, and therapeutic benefits in lupus nephritis (LN).

We initially investigated gene expression features in human kidney samples. By analyzing two transcriptome datasets (GSE112943: 14 LN patients and 7 healthy donors; GSE113342: 28 LN patients and 16 healthy donors) by GEO2R online tool, we found 85 overlapped differentially expressed genes (DEGs) between the two datasets, including 60 upregulated genes and 25 downregulated genes (Fig. 1a, b). Kyoto Encyclopedia of Genes and Genomes (KEGG) pathway enrichment showed that these DEGs were mainly involved in the type 17 T helper (Th17) cells differentiation (Fig. 1c).

**Fig. 1:**
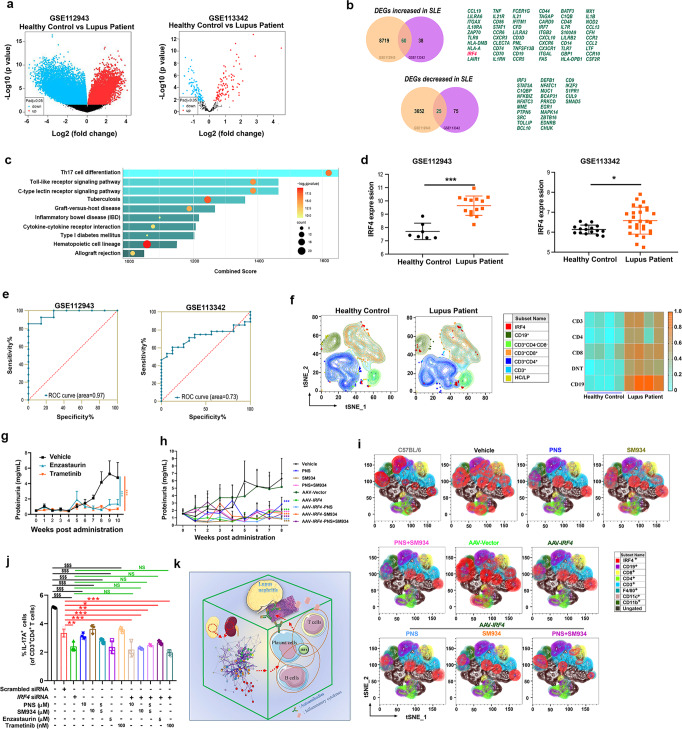
IRF4 expression is deeply involved in lupus nephritis and pathogenic immune responses in SLE. **a** Volcano plots display the DEGs in GSE112943 (left) and GSE113342 (right) datasets, comparing LN patients to healthy controls. **b** Venn diagrams showing the numbers of overlapping upregulated (upper) and downregulated (lower) DEGs between GSE112943 and GSE113342 sets. **c** KEGG pathway enrichment analysis of overlapping DEGs in GSE112943 and GSE113342 by Enrichr. **d** IRF4 expression in kidney tissues of LN patients and healthy controls (GSE112943 and GSE113342 sets). **e** The clinical sensitivity and specificity for IRF4 level were analyzed by ROC curves. **f** t-SNE plot of the B cell and T cell subsets overlaid with the IRF4 expression in PBMC of LN patients and healthy controls (left panel). Heatmap displayed the normalized IRF4 expression of indicated cell subsets (right panel). **g** Proteinuria level of the MRL/*lpr* mice treated with Enzastaurin or Trametinib. **h** Proteinuria level of MRL/*lpr* mice in different treatment groups. **i** Expression of IRF4, CD19, CD4, CD8, CD3, F4/80, CD11c, and CD11b on immune cells from indicated groups were mapped onto tSNE islands. **j** CD4^+^CD44^–^CD62L^+^ (naïve CD4^+^) T cells were differentiated under Th17-polarizing condition with indicated treatment, and the frequency of IL-17A^+^ cells were showed. **k** Graphical summary of relationship among the IRF4 expression, pathogenic T and B cells responses, autoantibodies overproduction and renal dysfunction in SLE

In an intervention experiment applying glucocorticoid prednisone (PNS) and the artemisinin derivative SM934 (β-aminoarteether maleate, molecular formula: C_21_H_33_NO_9_, a drug candidate for SLE treatment (Clinical trial: NCT03951259))^3^ that prevented progression of lupus nephritis (Supplementary Figs. S1–S4), we found that treatments altered gene expression pattern in kidneys of MRL/*lpr* mice, as revealed by the RNA-sequencing data on renal tissues (Supplementary Fig. S5). There were 34 downregulated and 3 upregulated genes in drug-treated groups vs. vehicle group (Supplementary Fig. S6). Of note, among the DEGs in MRL/*lpr* mice and human sample sets, *IRF4* was found as the sole common gene shared by the analytic sets displayed in Fig. 1b and Supplementary Fig. S6.

This finding prompted us to investigate the potential prognostic and therapeutic relevance of *IRF4* in LN. The elevation of renal *IRF4* expression in LN patients (Fig. 1d) and the area under receiver operating characteristic (ROC) curves (AUCs) (0.97 (*P* < 0.001) and 0.73 (*P* < 0.05), respectively for GSE112943 and GSE113342 dataset) (Fig. 1e) implied that IRF4 displays statistically significant accuracy as a marker to differentiate SLE patients from healthy individuals. Moreover, the level of renal IRF4 expression was strongly correlated with proteinuria, levels of anti-dsDNA and anti-cardiolipin (CL) antibodies, percentage of plasma cells, and germinal center (GC) B cells (*r* > 0.6), while moderately correlated with antinuclear antibody (ANA), IL-17A levels, and percentage of Th17 cells (0.4 < *r* < 0.59) in MRL/*lpr* mice (Supplementary Fig. S7). These findings suggested a closer link between renal IRF4 and B cell function in MRL/*lpr* mice than in LN patients. Corresponding with the finding in kidneys, the CD19^+^ B cells, CD3^+^CD4^+^ T cells, CD3^+^CD8^+^ T cells, and CD3^+^CD4^-^CD8^-^ double-negative (DN) T cells in spleens from aged MRL/*lpr* mice (Supplementary Fig. S8) and in peripheral blood mononuclear cells (PBMCs) from LN patients (Fig. 1f) displayed higher IRF4 expression, comparing to normal C57BL/6 mice and to healthy controls. Moreover, we observed improved lupus indications and attenuated IRF4 expression in kidneys of MRL/*lpr* mice treated with the reported inhibitors of IRF4,^4^ Trametinib and Enzastaurin (Fig. 1g and Supplementary Fig. S9). In light of this, an adoptive transfer experiment using severe combined immunodeficient (SCID) strain C.B-17 mice (recipient) and MRL/*lpr* mice (donor) was conducted. Unlike SCID mice transferred with *IRF4* intact cells, the ones transferred with AAV-sh*IRF4*-knockdown (AAV-*IRF4*) cells did not exhibit aggravating proteinuria, renal pathology or elevating circulating autoantibodies (Supplementary Fig. S10). Intriguingly, the AAV-*IRF4* MRL/*lpr* mice manifested much milder lupus symptoms, and these improvements were comparable to PNS and SM934 treatments, both in *IRF4* impaired or intact mice (Fig. 1h and Supplementary Fig. S11). A similar decrease of IRF4 expression in lymphocytes could also be observed in AAV-*IRF4* mice and in the aforementioned drug-treated mice (Fig. 1i).

To clarify whether the therapeutic effects of immunosuppressants and IRF4 inhibitors were relevant to the direct repression of IRF4 in immune cells, we conducted in vitro assays using splenocytes from MRL/*lpr* mice and normal C57BL/6 mice. Flow cytometry analysis demonstrated that PNS, SM934, Enzastaurin, and Trametinib abated IRF4 expression in T and B cells in a dose-dependent manner, rather than in macrophages or dendritic cells. However, IRF4 suppression was not present in cultures containing artemisinin and its derivatives (Supplementary Figs. S12–S14). Inconsistent with their in vivo effects (Supplementary Fig. S15), rapamycin displayed mild suppression of IRF4 on T cells, and methotrexate (MTX) slightly decreased IRF4 expression in B cells. Functionally, comparable inhibition of Th17 differentiation and IgGs production from LPS-stimulated CD19^+^ B cells were observed in cultures containing Enzastaurin, Trametinib, SM934, PNS, and SM934 + PNS combination (Fig. 1j and Supplementary Fig. S16). Particularly, *IRF4* ablation diminished Th17 differentiation, the suppression was equal to effects exhibited by SM934, PNS, Enzastautin and Trametinib (Fig. 1j and Supplementary Fig. S17).

Collectively, we proposed IRF4 as a competitive biomarker in SLE, especially in LN, based on the prominent expression of IRF4 in kidney tissue of SLE patients and lupus-prone mice and its tight link to disease progression and drug response. Albeit the correlation between autoantibodies and LN is clinically imprecise, our study indicated that ablating IRF4 expression in T and B lymphocytes could effectively ameliorate renal dysfunction and autoantibody overproduction respectively in SLE, thus providing a promising strategy for SLE treatment by restricting pathogenic T and B cells responses (Fig. 1k)

## Supplementary information

Supplementary Materials for Repression of Interferon Regulatory Factor-4 (IRF4) Hyperactivation Restricts Murine

Original and uncropped films of Western blots

## Data Availability

The RNA-sequencing data generated during this study have been deposited in the NCBI’s Gene Expression Omnibus (GSE112943 and GSE113342). Other data that support the findings of this study are available from the corresponding authors upon reasonable request.
