# Supplementary material for: Repression of interferon regulatory factor-4 (IRF4) hyperactivation restricts murine lupus
Source: Signal Transduct Target Ther. 2023 May 22;8:188. doi: 10.1038/s41392-023-01413-8 (PMC10200790; doi:10.1038/s41392-023-01413-8)
Supplement: Supplementary file 1 — Supplementary Materials for Repression of Interferon Regulatory Factor-4 (IRF4) Hyperactivation Restricts Murine [file 41392_2023_1413_MOESM1_ESM.docx]

Supplementary Materials for

Repression of Interferon Regulatory Factor-4 (IRF4) Hyperactivation Restricts Murine Lupus

Shijun He^1, 2, #, *^, Huihua Ding^3, #^, Li Chen^2, #^, Yiwei Shen ^3^, Yuting Liu^2^, Fenghua Zhu^2^, Xiaoqian Yang^2^, Nan Shen^3^, Zemin Lin^2, #, *^, Jianping Zuo^2, *^

Correspondence to: Shijun He (heshijun@shutcm.edu.cn, heshijun@simm.ac.cn); Zemin Lin (linzemin@simm.ac.cn); Jianping Zuo (jpzuo@simm.ac.cn)

^1^ Innovation Research Institute of Traditional Chinese Medicine, Shanghai University of Traditional Chinese Medicine, Shanghai, China;

^2^ Laboratory of Immunopharmacology, State Key Laboratory of Drug Research, Shanghai Institute of Materia Medica, Chinese Academy of Sciences, Shanghai, China;

^3^ Department of Rheumatology, Ren Ji Hospital, School of Medicine, Shanghai Jiao Tong University, Shanghai, China.

**This PDF file includes:**

Materials and Methods

Figures. S1 to S17

Materials and Methods

**Acquisition of Array Data and Processing**

We obtained the gene expression profiles of GSE112943 and GSE113342 in human kidney samples from LN patients and healthy controls from NCBI-GEO (<https://www.ncbi.nlm.nih.gov/geo/>).

The GEO2R (http://www.ncbi.nlm.nih.gov/geo/geo2r) online data analysis tool was used to compare the gene expression of the healthy controls and lupus patients. The original data was downloaded, the differentially expressed genes (DEGs) were screened, with the standard filter of *p*-values <0.05 and log|FC| ≥1.

**Disposition of mice**

MRL/MpJ-Fas^lpr^ (MRL/*lpr*), C.B-17 severe combined immunodeficient (C.B-17 SCID), and C57BL/6 mice were purchased from Shanghai SLAC Laboratory Animal Co., Ltd. All mice were maintained in a specific-pathogen-free (SPF) facility, and all animal studies were carried out in compliance with the institutional ethical guidelines on animal care and approved by the Institute Animal Care and Use Committee (IACUC) at Shanghai Institute of Materia Medica (SIMM).

To deplete IRF4 expression *in vivo*, AAV8 that contained *IRF4* shRNA (sh*IRF4*) and AAV-shControl was constructed by Genomeditech (Shanghai, China). The target sequence of sh*IRF4* was 5’- CCAACAAGCTAGAAAGAGA-3’. The sequence of negative control was 5’- TTCTCCGAACGTGTCACGT-3’. For AAV transduction, the viruses (2×10^11^ genomic copies/mouse) were delivered via tail vein injection to 8-week-old mice for 4 weeks, and then the transfected mice were orally gavaged with indicated drugs.

**Induction of lupus symptoms in SCID mice**

The CD19^+^ B cells and CD4^+^ T cells were enriched from AAV-sh*IRF4*-KD (AAV-*IRF4*) or AAV- shControl (AAV-vector) MRL/*lpr* mice aged at 12-week-old or from 8-week-old normal female C57BL/6 mice using FACSAria II (BD Biosciences, San Jose, USA). The resulted CD19^+^ B cells and CD4^+^ T cells were mixed in 1:10 ratio and then transferred into C.B-17 SCID mice at 3×10^7^ cell per mouse. All of the SCID mice were then maintained for 8 weeks with freely available food and water. At the end of the experiment, mice were anesthetized for serum and kidney collection.

**Drug treatment strategy**

Forty-five female MRL/*lpr* mice aged at 10-week-old were randomly divided into 5 groups as follows: vehicle (ddH_2_O), prednisolone (PNS) 2 mg/kg, SM934 10 mg/kg, PNS (1 mg/kg) + SM934 H (10 mg/kg), PNS (1 mg/kg) + SM934 L (5 mg/kg) (n=9 per group). Mice were orally gavaged with indicated drug once a day for 18 weeks. Urine from individual mice was collected weekly and the concentration of urinary protein was detected. At the end of the experiment, mice were anaesthetized for serum collection. Lymph nodes, spleens, kidneys, and skin were isolated and analyzed.

Forty-five female MRL/*lpr* mice aged at 16-week-old were used. Twenty *IRF4* intact mice were randomly divided into 4 groups treated with indicated drugs as follows: vehicle (ddH_2_O), PNS 2 mg/kg, SM934 10 mg/kg, PNS 1 mg/kg + SM934 5 mg/kg (n=5 per group). Twenty-five mice were injected with AAV-sh*IRF4* (AAV-*IRF4*) or AAV-shControl (AAV-vector) and divided into 5 groups and treated with indicated drugs as follows: AAV-vector (ddH_2_O), AAV-*IRF4* (ddH_2_O), AAV-*IRF4-*PNS (PNS 2 mg/kg), AAV-*IRF4-*SM934 (SM934 10 mg/kg), AAV-*IRF4-*PNS + SM934 (PNS 1 mg/kg + SM934 5 mg/kg) (n=5 per group). All of the 45 mice were orally gavaged with indicated drug once a day for 8 weeks. Urine from individual mice was collected weekly and the concentration of urinary protein was detected.

Thirty female MRL/*lpr* mice and ten female C57BL/6 mice aged at 10-week-old were used. MRL/*lpr* mice were randomly divided into 3 groups treated with indicated drugs as follows: vehicle (ddH_2_O), Enzastaurin (75 mg/kg), Trametinib (3 mg/kg) (n=10 per group). C57BL/6 mice were administered with ddH_2_O. Mice were orally gavaged with indicated drug once a day for 10 weeks. Urine from individual mice was collected weekly and the concentration of urinary protein was detected.

Thirty female MRL/*lpr* mice and ten female C57BL/6 mice aged were used. MRL/*lpr* mice were randomly divided into 3 groups treated with indicated drugs as follows: vehicle (ddH_2_O), methotrexate (MTX, 1 mg/kg), rapamycin (1 mg/kg) (n=10 per group). C57BL/6 mice were administered with ddH_2_O. Mice were orally gavaged with indicated drug once a day for 5 weeks. Urine from individual mice was collected weekly and the concentration of urinary protein was detected.

**Flow cytometric analysis**

Antibodies for surface staining: FITC-conjugated anti-m (mouse) CD3e (145-2C11), Percp-cy5.5-conjugated anti-mCD3e (145-2C11), BUV395-conjugated anti-mCD4 (GK1.5), BUV737-conjugated anti-mCD4 (RM4.5), FITC-conjugated anti-mCD8a (53-6.7), APC-conjugated anti-mCD8a (53-6.7), APC-H7-conjugated anti-mCD19 (1D3), BV510-conjugated anti-mB220 (RA3-6B2), BV421-conjugated anti-mCD138 (281-2), BV395-conjugated anti-mCD11b (M1/70), BV510-conjugated anti-mF4/80 (T45-2342), APC-conjugated anti-mCD11c (HL3), BV421-conjugated anti-mIL-17A (TC11-18H10), PE-conjugated mIL-17A (TC11-18H10), BV421-conjugated anti-h (human) CD3 (SK7), Percp-cy5.5-conjugated anti-hCD4 (SK3), PE-conjugated anti-hCD8 (SK1), BV510-conjugated anti-hCD19 (SJ25C1), PE-conjugated anti-h/mIRF4 (Q9-343) were purchased from BD Bioscience. FITC-conjugated anti-h/mIRF4 (3E4) was purchased from eBioscience. Fixable Viability Dye (eFluor 780) purchased from eBioscience was used to gate live cells. The FoxP3 Staining Buffer set (eBioscience) was used to fix and permeabilize cells for intracellular staining of IL-17A and IRF4. All measurements were performed on 4-laser/13-color BD LSRFortessa (BD Biosciences, San Jose, USA) and results were analyzed using FlowJo software (Tree Star, Inc., Ashland, OR, USA).

***In vitro* evaluation of IRF4 in cellular culture**

Splenocytes isolated from 8-10-week-old female MRL/*lpr* mice were incubated with PNS, SM934 or inhibitors under stimulation of PMA (1 μg/ml) and ionomycin (1 μM) to determine the expression of IRF4 in T and B cells.

Splenocytes isolated from 8-week-old female C57BL/6 mice were incubated with artemisinin, artemisinin derivatives, PNS, or indicated immunosuppressants under stimulation of PMA (1 μg/ml) and ionomycin (1 μM) to determine the expression of IRF4 in T and B cells.

***In vitro* Th17 cell differentiation**

Naïve CD4^+^ (CD4^+^CD44^–^CD62L^+^) T cells were enriched by using FACSAria II (BD Biosciences). 1×10^6^/well resulted cells were activated with anti-CD3 mAb (clone 2C11, 5 μg/mL) and anti-CD28 mAb (clone 37.51, 2 μg/ml) and were induced to indicated type of T helper cells as we previous reported ^1^. Briefly, murine IL-6 (mIL-6; 25 ng/ml, BD PharMingen), hTGFβ1 (10 ng/ml, Peprotech), mIL-23 (20 ng/ml; eBioscience), anti-mIFNγ mAbs (clone XMG1.2, 10 μg/mL, eBioscience), and anti-mIL-4 (clone 11B11, 10 μg/mL, eBioscience) were added to the cultures to induced Th17 differentiation.

**IRF4 depletion in naïve CD4^+^ T cells**

Naïve CD4^+^ (CD4^+^CD44^–^CD62L^+^) T cells from female C57BL/6 mice were enriched by using FACSAria II (BD Biosciences). The purity of the resulting Naïve CD4^+^ T cell populations was examined by flow cytometry analysis and was consistently >98%. To knock-down the IRF4 expression in Naïve CD4^+^ T cells, siRNA targeting for *IRF4* silence (Genomeditech, Shanghai, China) was used following the manufacturer’s instructions. Briefly, cells were transfected with siRNA, mixed with Lipofectamine® RNAiMAX Reagent (Thermo Fisher Scientific, MA, USA) in serum-free Opti-MEM medium (Thermo Fisher Scientific, NY, USA). The target sequence of siRNAs for IRF4 was 5’- CCAACAAGCTAGAAAGAGA-3’.

**Measurements of urinary protein**

Urine from lupus-prone mice was collected at indicated time. Proteinuria was measured using Bradford's method using Coomassie Brilliant Blue R-250 staining solution (Bio-Rad Laboratories, CA, USA). Urinary creatinine was measured using a commercial assay kit from Nanjing Jiancheng Bioengineering Institute (Nanjing, China), according to the manufacturer’s instructions.

**Gross pathology**

Skin pathology and lymphadenopathy were evaluated weekly as reported ^2, 3^. Briefly, the lupus-erythematosus-like skin lesions for changes in the ears, nose, and interscapular region were scored based on the number of lesions and area, using a scoring system as following: 0, none; 1, mild lesions (< 0.5 cm); 2, moderate lesions (two or more, < 2 cm); 3, severe lesions (multiple, ≧2 cm). Lymphadenopathy (cervical, brachial, and inguinal) was scored from 0 to 3 based on the number and size of palpable nodes. 0, none; 1, one node; 2, two small-to-moderate nodes; 3, three or more moderate-to-large nodes. Body weight was measured weekly. Splenomegaly was determined by spleen weight. At the end of the treatment period, spleen and kidney weight were determined. The Spleen index was determined as the ratio of spleen weight to body weight.

**Tissue histology**

Skin samples were collected from the back of the neck of mice. Both of skin and kidney tissues were fixed in 4% formalin and embedded in paraffin for the histopathological examination. Three-micrometer-thick sections were prepared for H&E (hematoxylin and eosin), PAS (periodic acid–Schiff) and Masson’s trichome staining. Microscopy was performed acquired by DM RXA2 (Leica Microsystems AG, Wetzlar, Germany).

**ELISA**

Levels of serum antibodies antinuclear antibodies (ANAs), anti-double-stranded DNA (anti-dsDNA) Abs and anti-cardiolipin (anti-CL) antibodies were detected by ELISA as we previously reported ^4^.

**Multiplex assay/suspension bead array**

Serum of MRL/*lpr* mice was analyzed using a Luminex multiplex assay-based suspension bead array (SBA) by a ProcartaPlex Assay kit EPX170-26087-901 (Invitrogen, Carlsbad, CA, USA). Supernatants from CD19^+^ B cell culture stimulated with/without LPS (10 μg/mL) for 120 hours in the presence or absence Enzastaurin or Trametinib was analyzed using a Luminex multiplex assay-based SBA by a ProcartaPlex mouse antibody isotyping panel 7-plex assay kit (EPX070-20815-901, Invitrogen). Assay plates were read by Luminex 200 Instruments (Luminex, Austin, TX). The concentrations were calculated by the xPONENT software (LUMINEX, Austin, TX, USA). Circular heatmap was plotted using an online platform for data analysis and visualization (https://www.bioinformatics.com.cn).

**RNA sequencing**

Mice kidneys were snap-frozen in liquid nitrogen. Kidney cortex tissues of each group were sent to Majorbio Bio-Pharm Technology Co. Ltd. (Shanghai, China) for RNA isolation and sequencing. The data were analyzed using I-Sanger Cloud Platform ([www.i-sanger.com](http://www.i-sanger.com)).

**Analysis of human PBMCs**

PBMCs from treatment-naive LN patients (all LN patients had a renal Systemic Lupus Erythematosus Disease Activity Index (rSLEDAI) equal or more than 4) and age- and gender-matched healthy controls were collected and studied in this research.

Informed consent was obtained from all the participants before the study. The study was approved by the Ethics Committee of Renji Hospital, Shanghai JiaoTong University School of Medicine, Shanghai, China. PBMCs were isolated from heparinized blood by Ficoll-Hypaque density gradient centrifugation (Pharmacia Biotech, Sweden) and analyzed by flow cytometry.

**Statistical analysis**

All experiments were performed using 5-10 mice per group or repeated at least 3 times and done in triplicate. Unpaired 2-tailed t-test was performed for comparisons between 2 groups in pairwise comparisons. One-way analysis of variance (ANOVA) followed by Turkey’s multiple comparison test is applied in detecting differences across three or more groups. Mice survival experience was visualized by Kaplan–Meier curves and statistically assessed by log-rank (Mantel-Cox) test. The Pearson correlation coefficient evaluate the correlation between IRF4 expression and lupus disease clinical manifestations. Receiver operating characteristic curves (ROC) was done by plotting sensitivity on the y-axis and 100%-specificity on the x-axis. Statistical analysis was performed using GraphPad Prism 8.1(GraphPad Software, San Diego, CA, USA) statistical software. P values less than 0.05 were considered significantly.

**References**

1 Hou, L. F. *et al.* Oral administration of artemisinin analog sm934 ameliorates lupus syndromes in mrl/lpr mice by inhibiting th1 and th17 cell responses. *Arthritis Rheum*, **63**: 2445-2455, (2011).

2 Kinoshita, K. *et al.* Blockade of il-18 receptor signaling delays the onset of autoimmune disease in mrl-faslpr mice. *J Immunol*, **173**: 5312, (2004).

3 Swenson, J., Olgun, S., Radjavi, A., Kaur, T. & Reilly, C. M. Clinical efficacy of buprenorphine to minimize distress in mrl/lpr mice. *Eur J Pharmacol*, **567**: 67-76, (2007).

4 He, S. J. *et al.* Reversible sahh inhibitor protects against glomerulonephritis in lupus-prone mice by downregulating renal alpha-actinin-4 expression and stabilizing integrin-cytoskeleton linkage. *Arthritis Res Ther*, **21**: 40, (2019).

Figures S1-S17

Figure. S1.


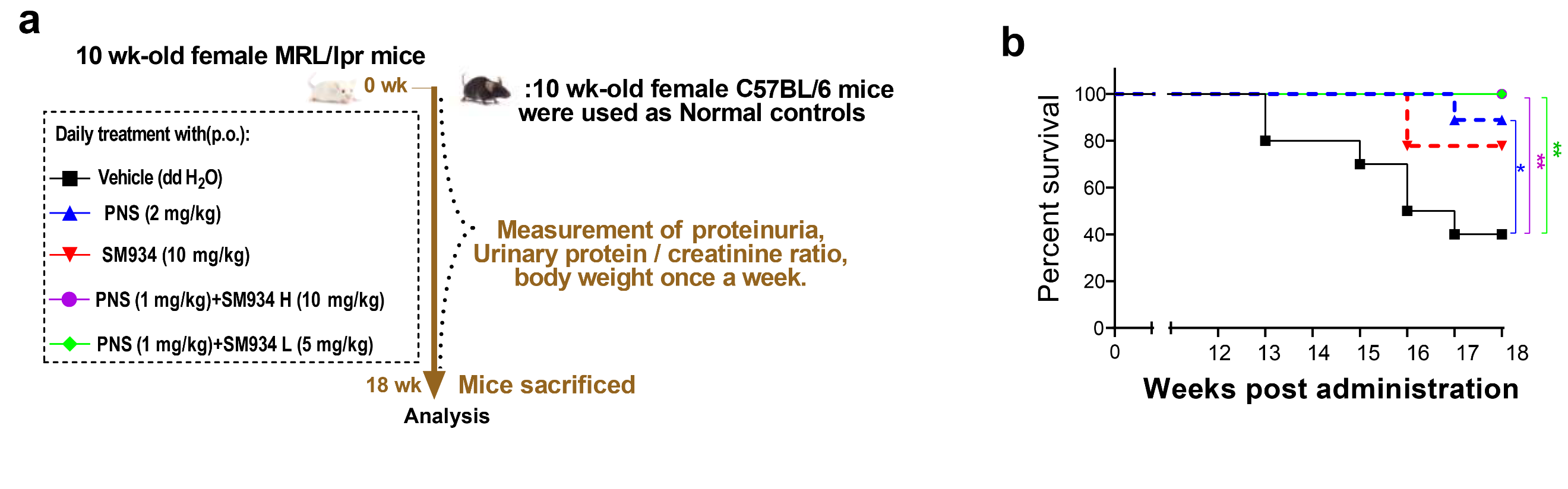


**Fig. S1. Intervention experiment in MRL/*lpr* mice with SM934 and PNS.** MRL/*lpr* mice aged at 10-week-old were randomly divided into 5 groups as follows: vehicle (ddH_2_O), prednisolone (PNS) 2 mg/kg, SM934 10 mg/kg, PNS (1 mg/kg) + SM934 H (10 mg/kg), PNS (1 mg/kg) + SM934 L (5 mg/kg) (n=9 per group). Mice were orally gavaged with indicated drug once a day for 18 weeks. Schematic of experimental protocol (a) and Kaplan-Meier survival analysis (b) of the MRL/*lpr* mice treated with ddH2O, PNS, SM934 or their combinations.

Figure. S2.

**
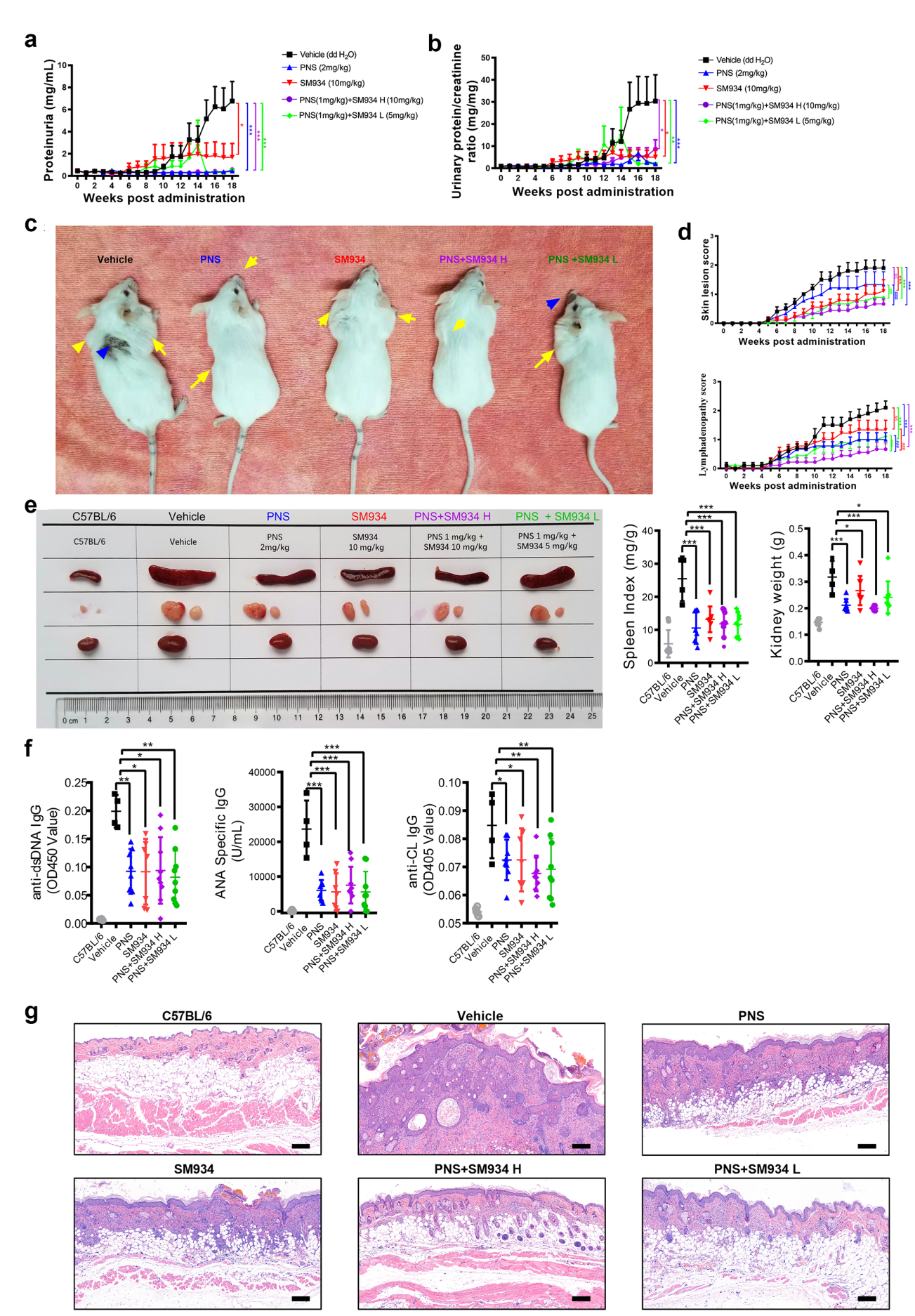
**

**Fig. S2. SM934 and PNS alone and their combination attenuated lupus symptoms in MRL/*lpr* mice.** (a, b) The proteinuria level and urinary protein/ creatinine ratio were monitored once a week. (c) Features of dermatitis and lymphadenopathy. (d) The severity of dermatitis and lymphadenopathy were graded by a semiquantitative scale once a week. (e) The spleens, lymph nodes, and kidneys of the MRL/*lpr* mice in each group were observed (left panel) and spleen weight and kidney weight were measured at the end of the treatment. Spleen index (mg/g) = (Spleen weight/Mice weight) × 1000. (f) Levels of serum anti-dsDNA autoantibodies, ANA-specific IgG, and anti-CL IgG. (g) Representative images of showing histological features of dermatitis detected by H&E staining (scale bars: 200 μm). n=4-9 per group. Data were represented as mean ± SD. * P<0.05, ** P<0.01, *** P<0.001 versus the vehicle group.

Figure. S3.

**Z
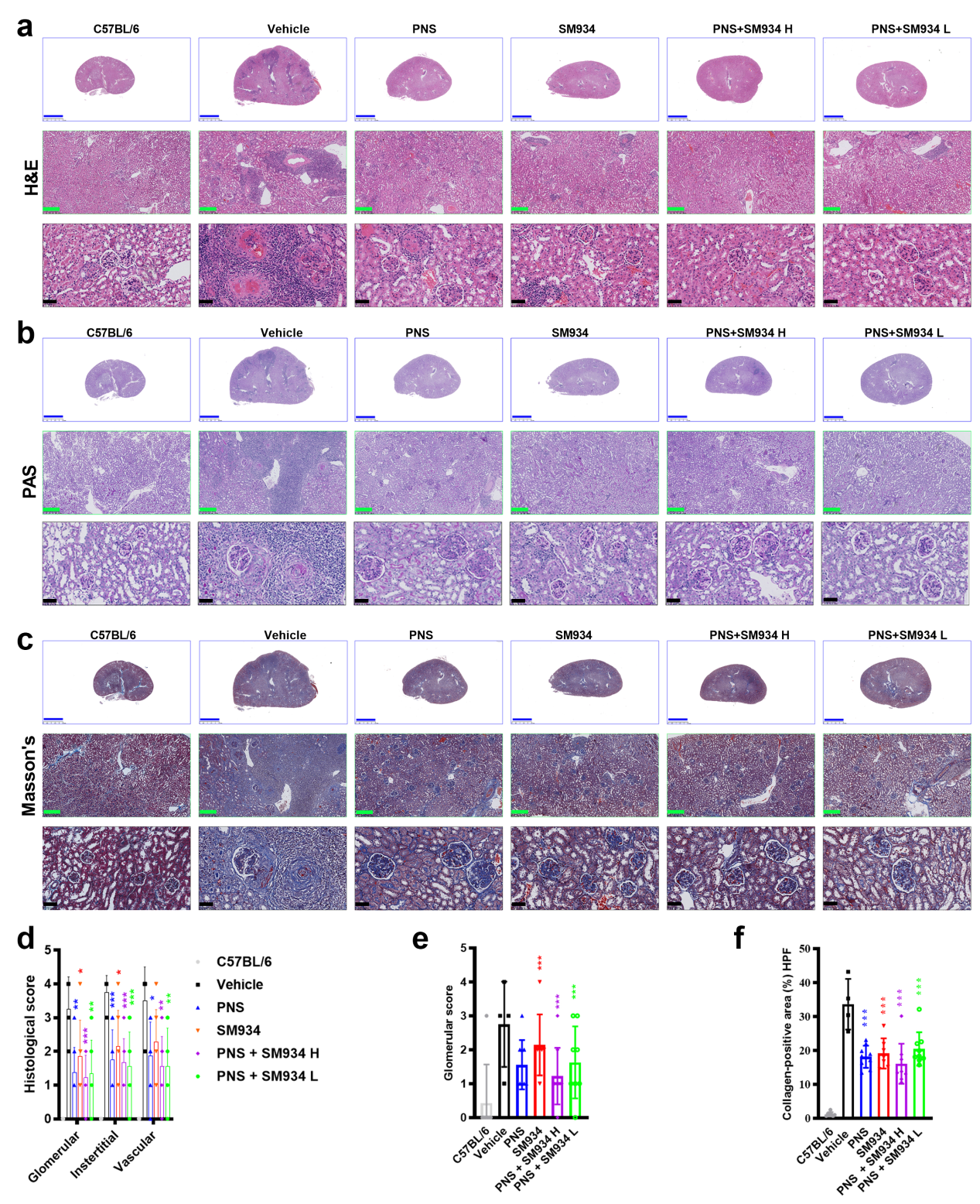
**

**Fig. S3. SM934 and PNS alone and their combination improved renal pathology in MRL/*lpr* mice.** Mice were orally gavaged with indicated drug once a day for 18 weeks. Representative images showing pathological changes of kidneys detected by H&E (H&E; a, d), Periodic Acid-Schiff (PAS; b, e) and Masson’s trichrome (Masson’s; c, f). Scale bars: top, 2.5 mm; middle, 250 μm; bottom, 50 μm (a, b, c). n=4-9 per group. Data were represented as mean ± SD. * P<0.05, ** P<0.01, *** P<0.001 versus the vehicle group.

Figure. S4.

**
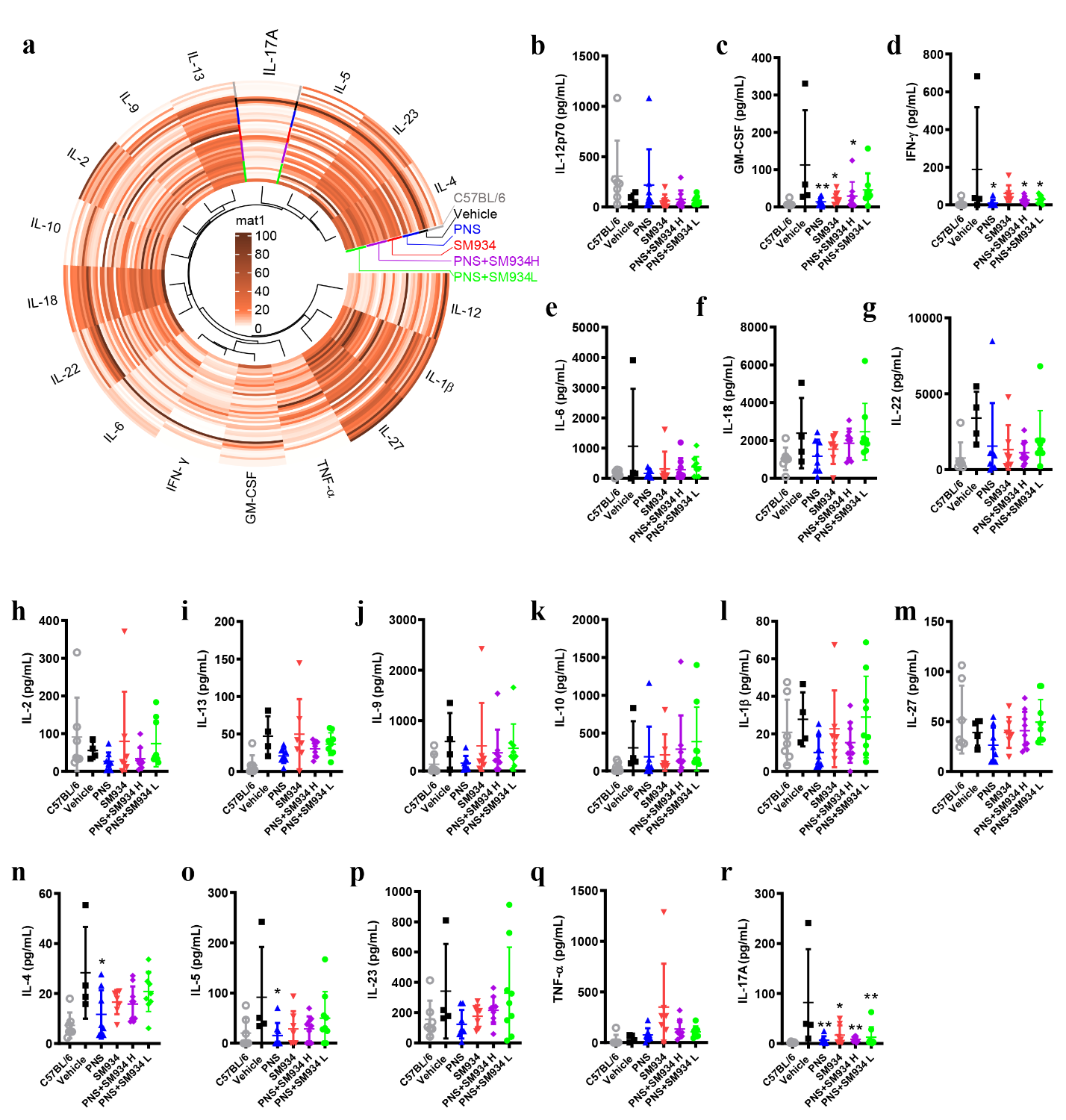
**

**Fig. S4.** **Cytokine levels in terminal serum of C57BL/6 mice and MRL/*lpr* mice.** (a) Circular heatmap of 17 cytokines concentration in serum of C57BL/6 mice and MRL/*lpr* mice under different treatments detected by ProcartaPlex Assay kit. Vehicle (ddH_2_O), prednisolone (PNS) 2 mg/kg, SM934 10 mg/kg, PNS (1 mg/kg) + SM934 H (10 mg/kg), PNS (1 mg/kg) + SM934 L (5 mg/kg). n=4-9 per group. (b-r) Statistic data were represented as mean ± SD. * P<0.05, ** P<0.01, versus the vehicle group.

Figure. S5.

**
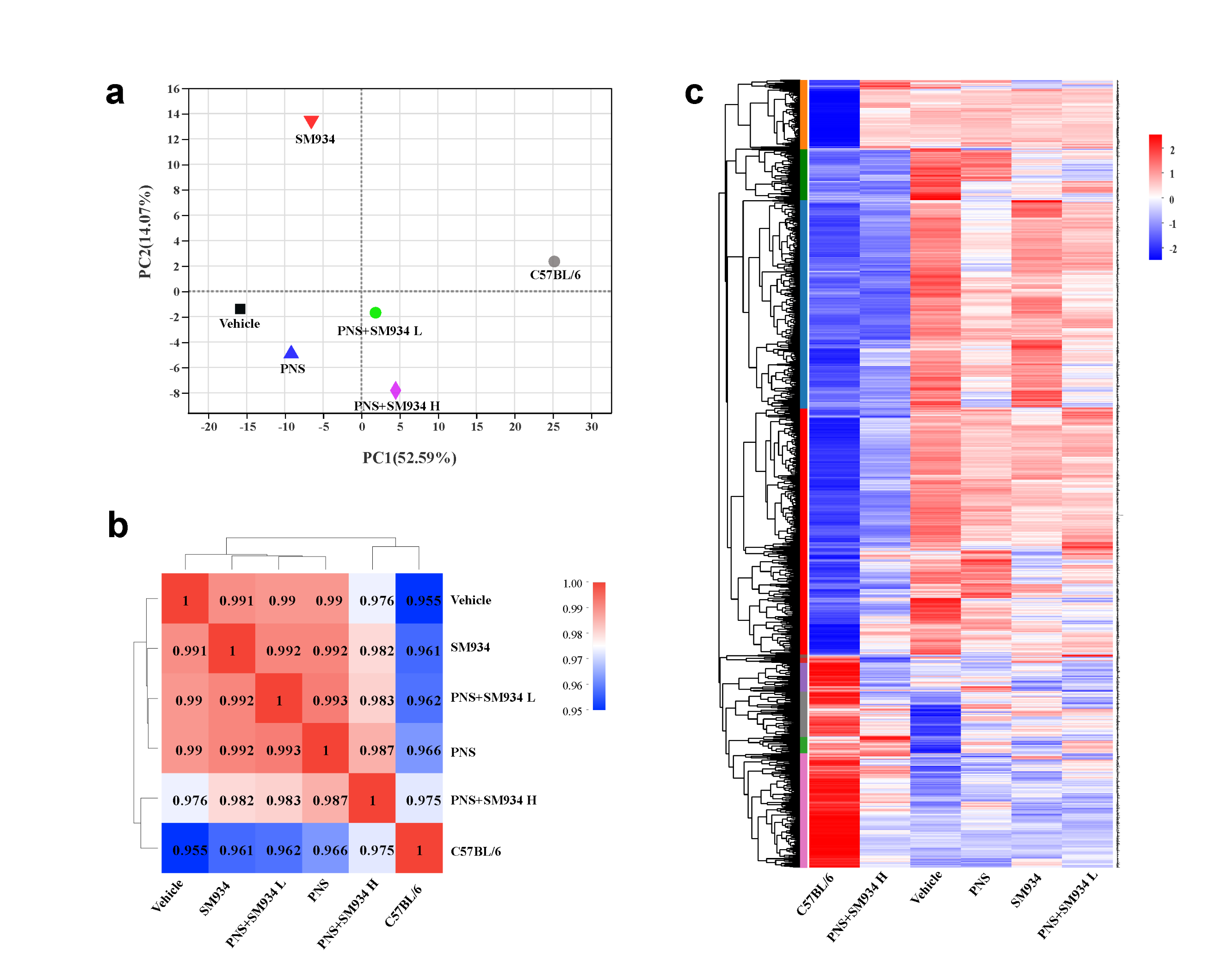
**

**Fig. S5.** **RNA-sequencing (Seq) data derived from MRL/*lpr* mice.** (a) Principal component analysis of the RNA-seq data of kidney tissues. (b) Correlation matrix of combined samples under indicated treatment conditions, based on Pearson correlation coefficients. (c) Hierarchical clustering heat map of genes affected significantly by all the therapy treatment groups. Colored bar scale indicates the standardized gene expression values. n=4-9 per group.

Figure. S6.


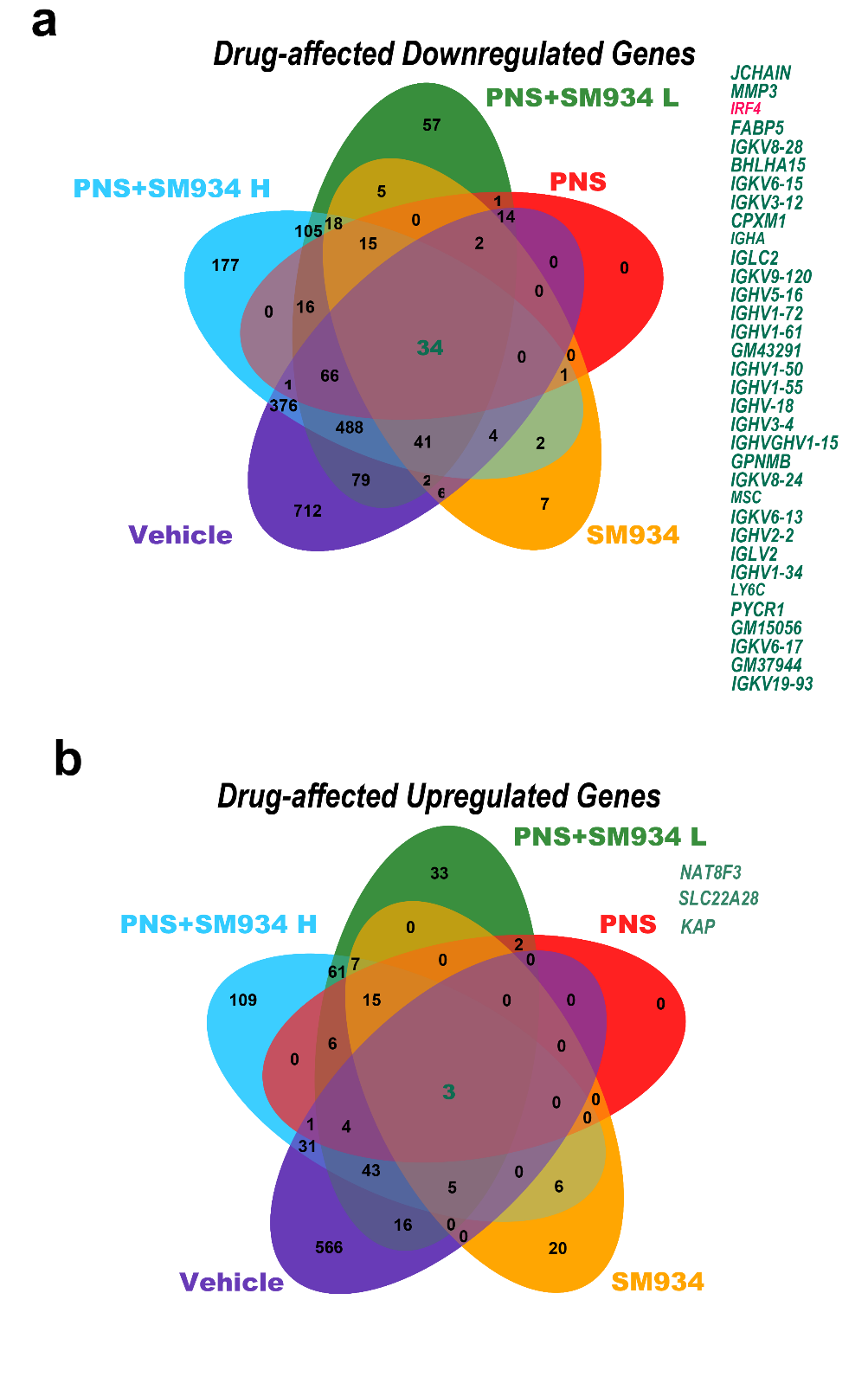


**Fig. S6.** **Drug-affected genes among kidney tissues from drug or vehicle-treated MRL/*lpr* mice.** Venn diagram showing the numbers of overlapping downregulated (a) and upregulated (b) DEGs among kidney tissues from PNS, SM934 or their combinations-treated MRL/*lpr* mice. n=4-9 per group.

Figure. S7.


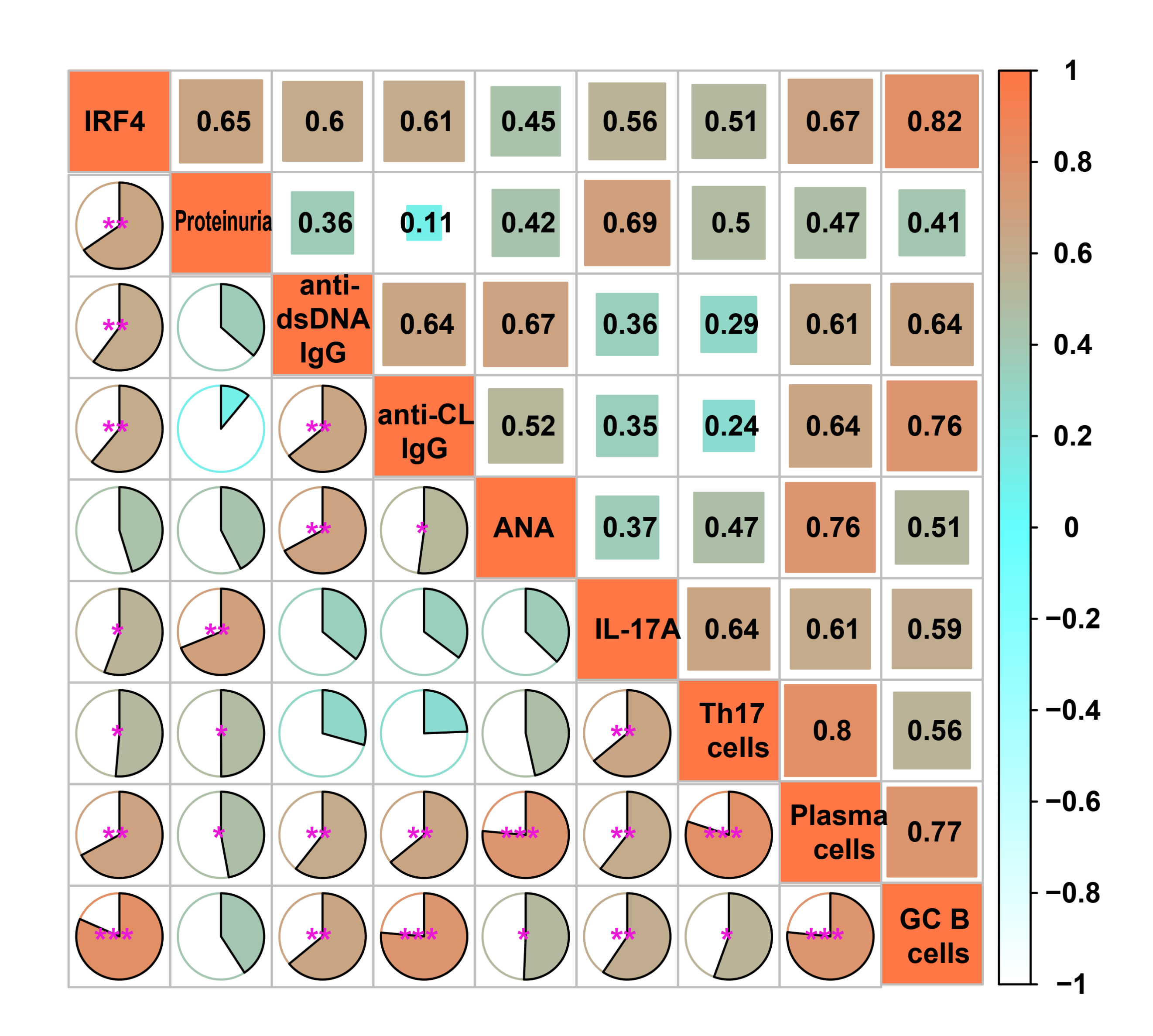


**Fig. S7.** **Correlation analysis between renal IRF4 and lupus-associated indices in MRL/*lpr* mice.** Correlation matrix heatmap displays correlation between renal IRF4 expression and proteinuria, serum IL-17A, serum anti-dsDNA IgG, serum anti-CL IgG, serum ANA, splenic plasma cells proportion, and Th17 proportion in MRL/*lpr* mice. Significance marks and the correlation coefficient. n=18.

Figure. S8.


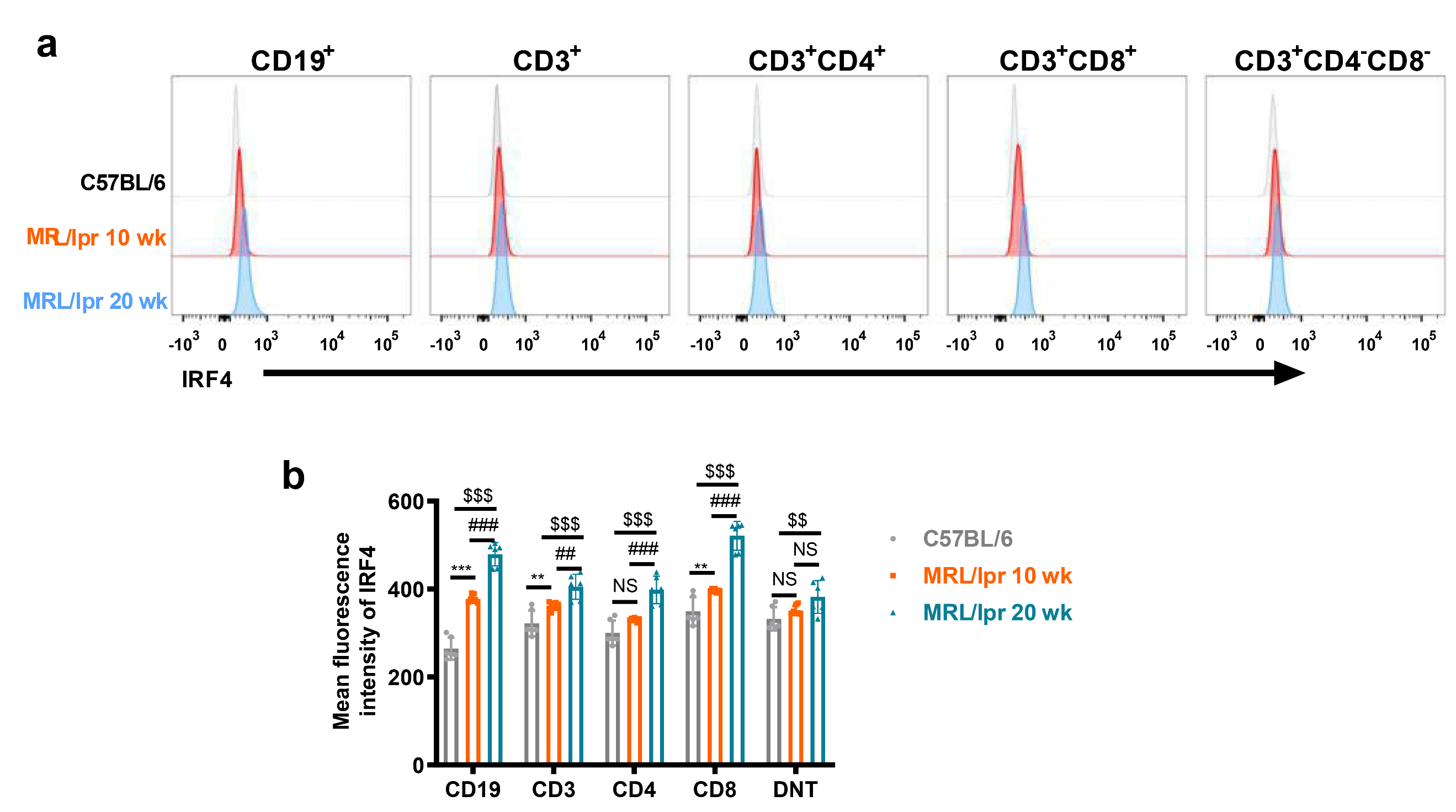


**Fig. S8.** **Level of IRF4 expression in B cell and T cell subsets of normal and lupus-prone mice at indicated ages.** Representative results of flow cytometric diagram (a) and statistical analysis (b) of IRF4 expression in B cell and T cell subsets of normal and lupus-prone mice at indicated ages. n=6 per group. Statistic data were represented as mean ± SD. **/^##^/^$$^ P<0.01, ***^/###/$$$^ P<0.001.

Figure. S9.


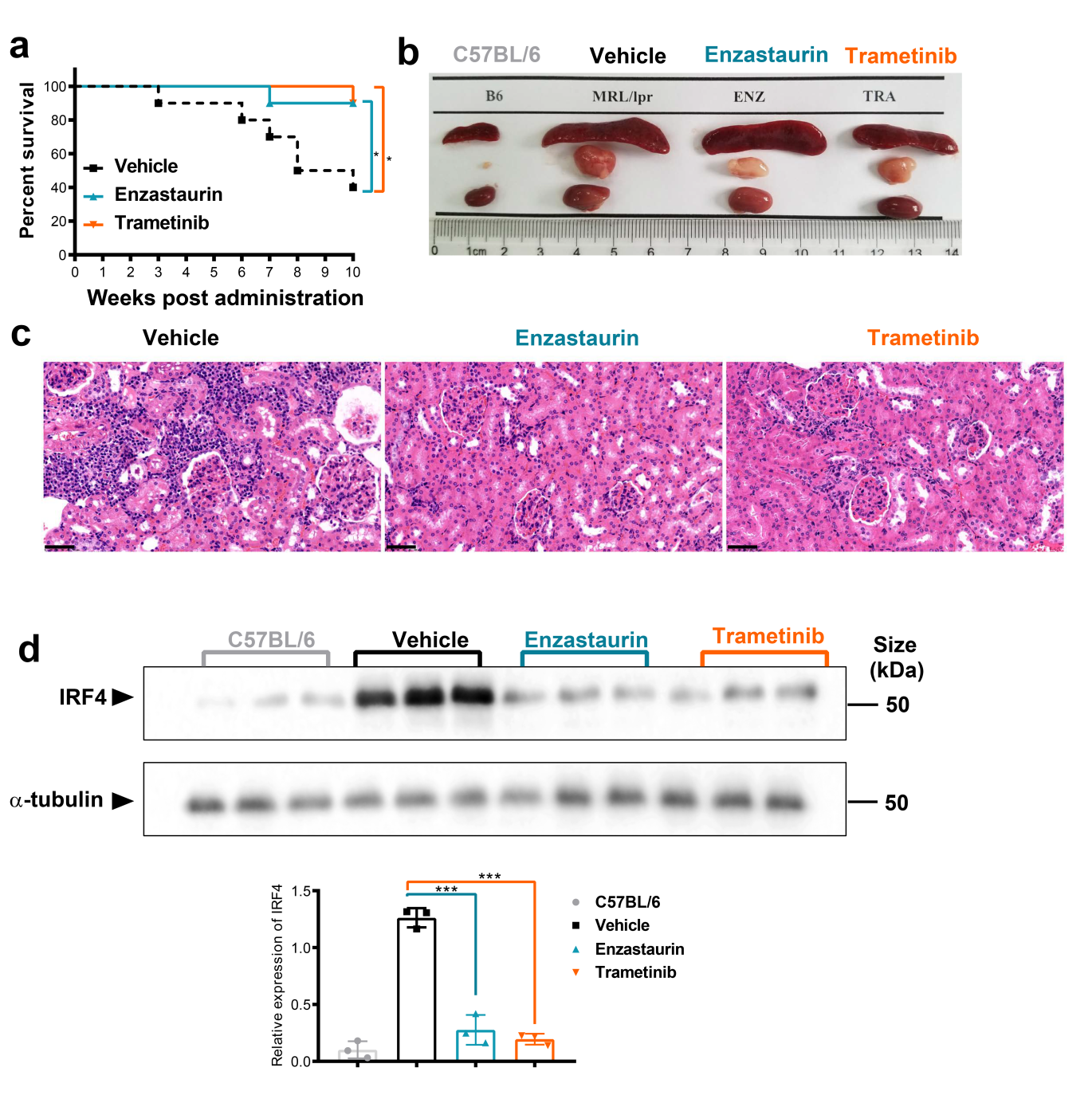


**Fig. S9.** **IRF4 inhibitors ameliorated lupus and suppressed renal IRF4 expression in MRL/lpr mice.** (a) Kaplan-Meier survival analysis of the MRL/*lpr* mice treated with Enzastaurin or Trametinib. n=10 per group. (b) Representative photos of spleens, lymph nodes, and kidneys of the MRL/lpr mice in each group. (c) Photomicrographic representation of kidney glomeruli detected by H&E staining (scale bars: 50 μm). (d) Representative Western blot and quantification analysis of IRF4 and α-tubulin in kidney tissue. α-tubulin protein was used as the loading control for the semi-quantification of IRF4 levels. n=3. Statistic data were represented as mean ± SD. *** P<0.001 versus the vehicle group.

Figure. S10.


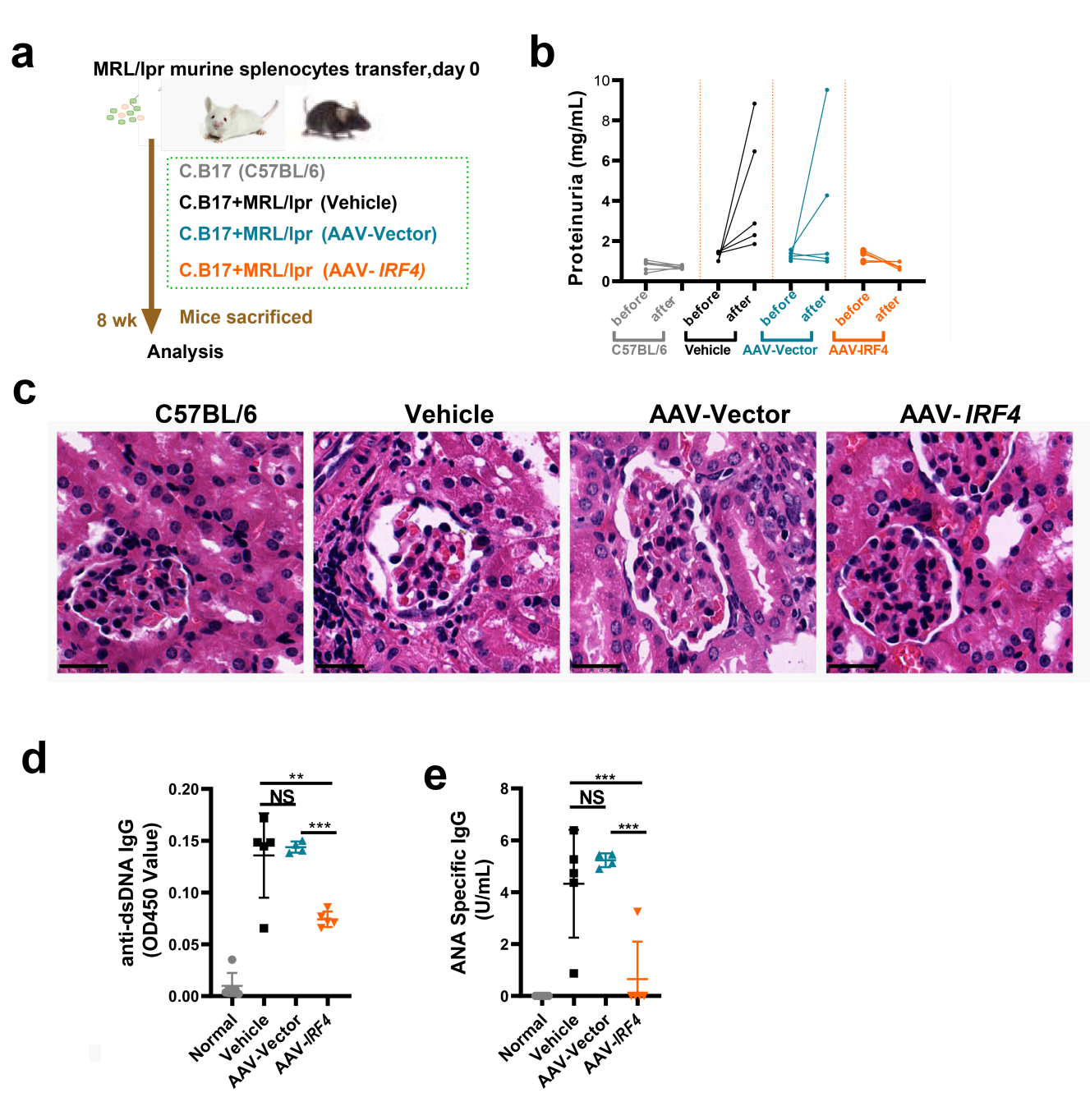


**Fig. S10. SCID mice transferred AAV-*IRF4* cells manifested milder lupus symptoms.** Schematic of experimental protocol (a), proteinuria level (b), photomicrographic representation of kidney glomeruli detected by H&E staining (c) and levels of serum anti-dsDNA autoantibodies (d) and ANA-specific IgG (e) of the SCID mice transferred with indicated immune cells (scale bars: 25 μm). n=5-6 per group. Statistic data were represented as mean ± SD. ** P<0.01, *** P<0.001 versus the vehicle group.

Figure. S11.


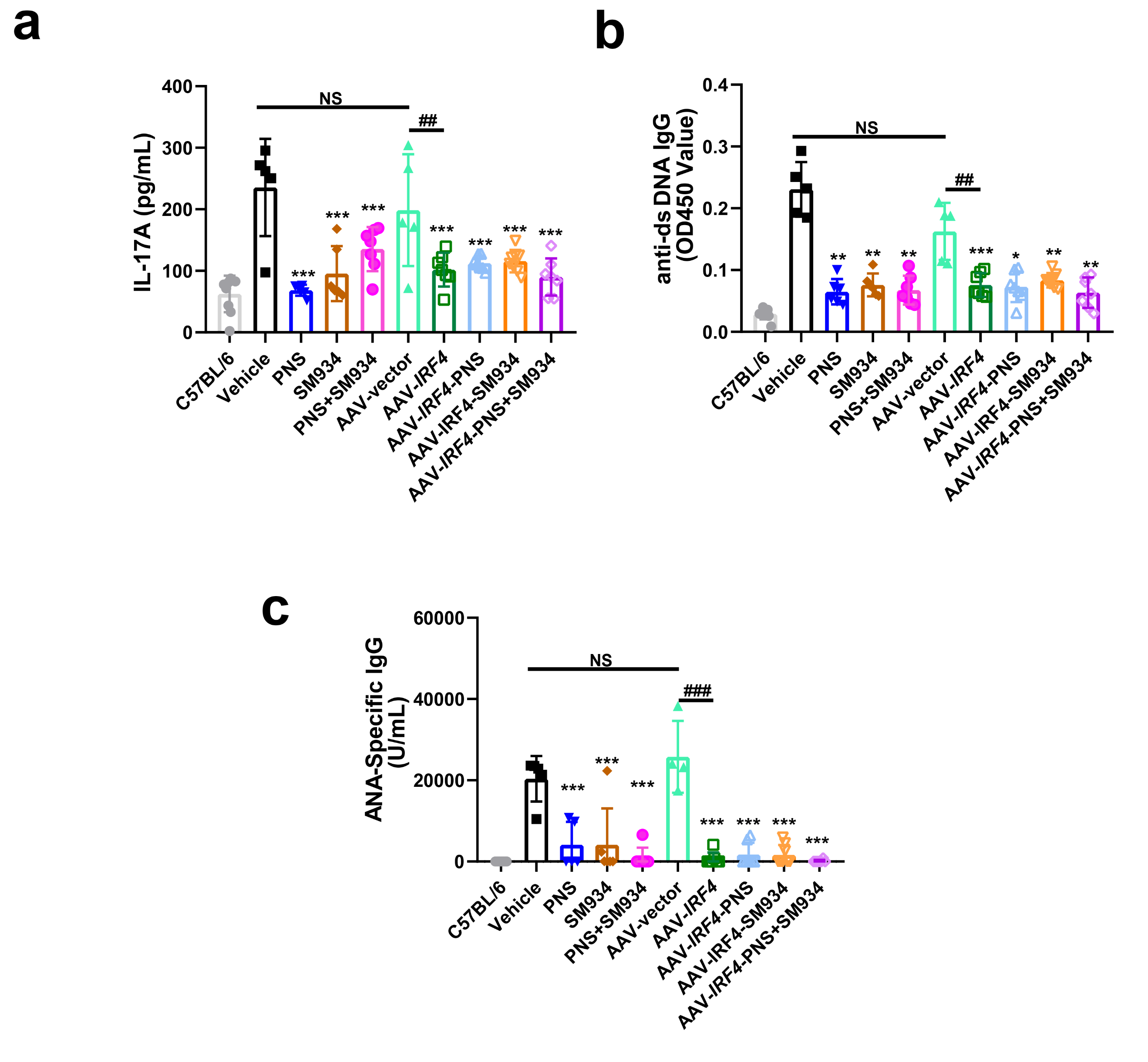


**Fig. S11. *IRF4* knockdown resulted in attenuated lupus symptoms in MRL/*lpr* mice.** Levels of serum IL-17A (a), anti-dsDNA autoantibodies (b), and ANA-specific IgG (c). n=4-8 per group. Statistic data were represented as mean ± SD. * P<0.05, ** P<0.01, *** P<0.001.

Figure. S12.


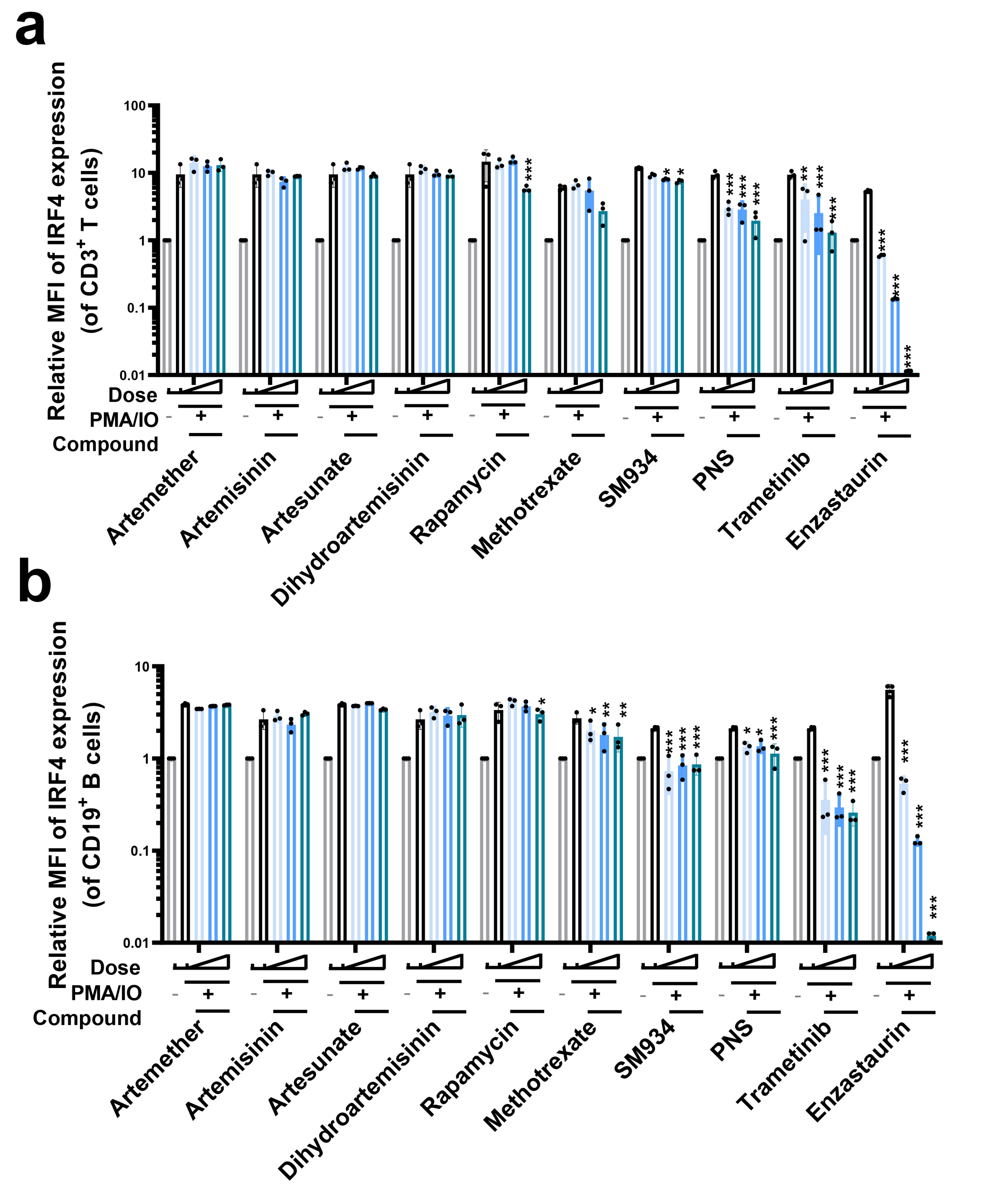


**Fig. S12. PNS, SM934, Enzastaurin, and Trametinib directly abated IRF4 expression in T and B cells of normal mice.** Expression of IRF4 on CD3^+^ T cells (a) and CD19^+^ B cells (b) in the *in vitro* cultures of splenocytes from C57BL/6 mice. Cells were treated with indicated compounds and stimulated by 1 µg/mL phorbol myristate acetate (PMA) and 1 µM ionomycin (IO) for 24 h at 37°C and 5% CO_2_. n=3 per group. Statistic data were represented as mean ± SD. * P<0.05, ** P<0.01, *** P<0.001.

Figure. S13.


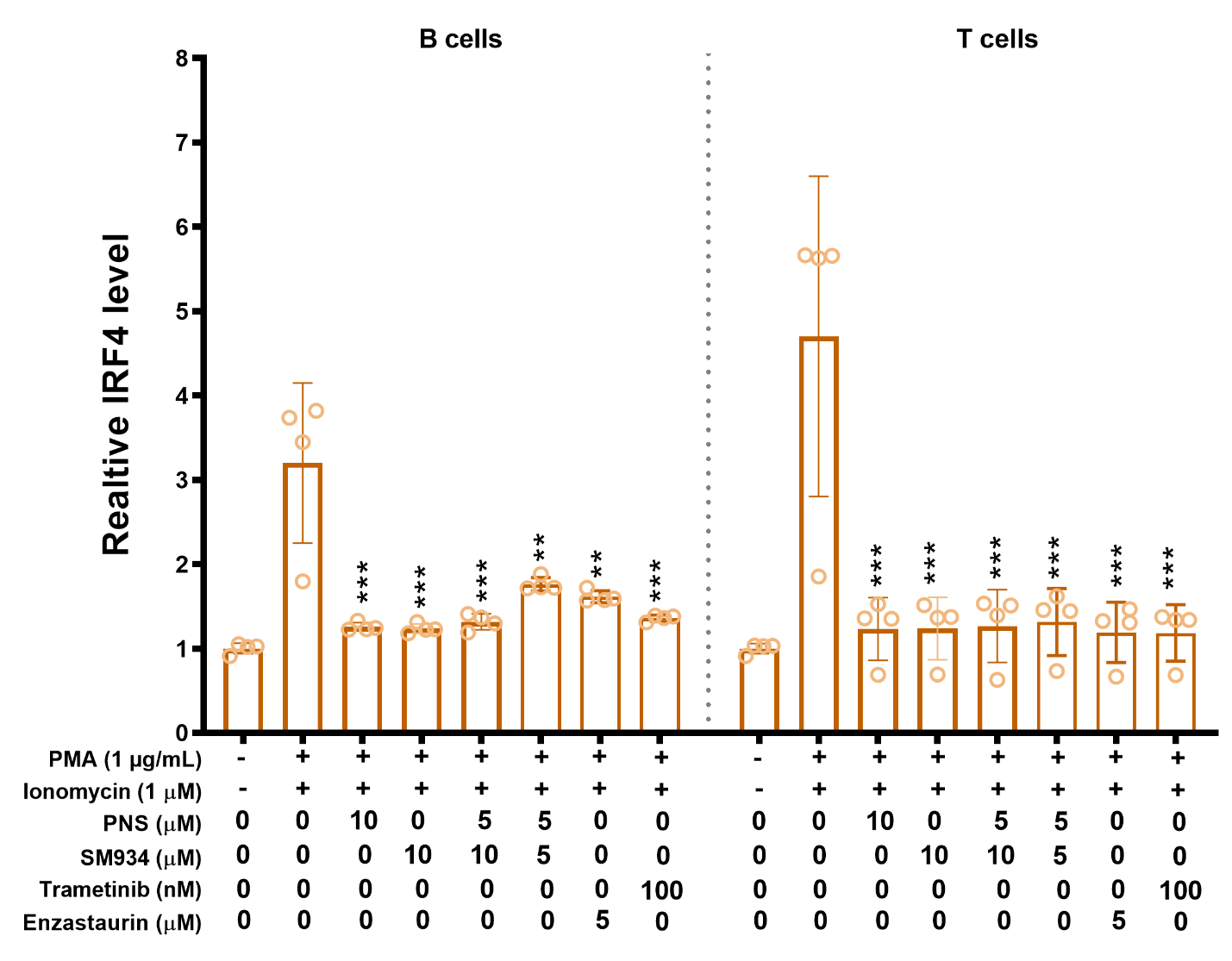


**Fig. S13. PNS, SM934, Enzastaurin, and Trametinib directly suppressed IRF4 expression in T and B cells of MRL/*lpr* mice.** Relative expression of IRF4 in CD19^+^ B cells and CD3^+^ T cells of the spleens of MRL/*lpr* mice. Cells were treated with the indicated compounds for 24 hours in the presence of 1 µg/mL phorbol myristate acetate (PMA) and 1 µM ionomycin (IO) at 37°C and 5% CO2. ** P<0.01, *** P<0.001 versus cells only cultured with PMA and IO; n=4 per group. Statistic data were represented as mean ± SD.

Figure. S14.
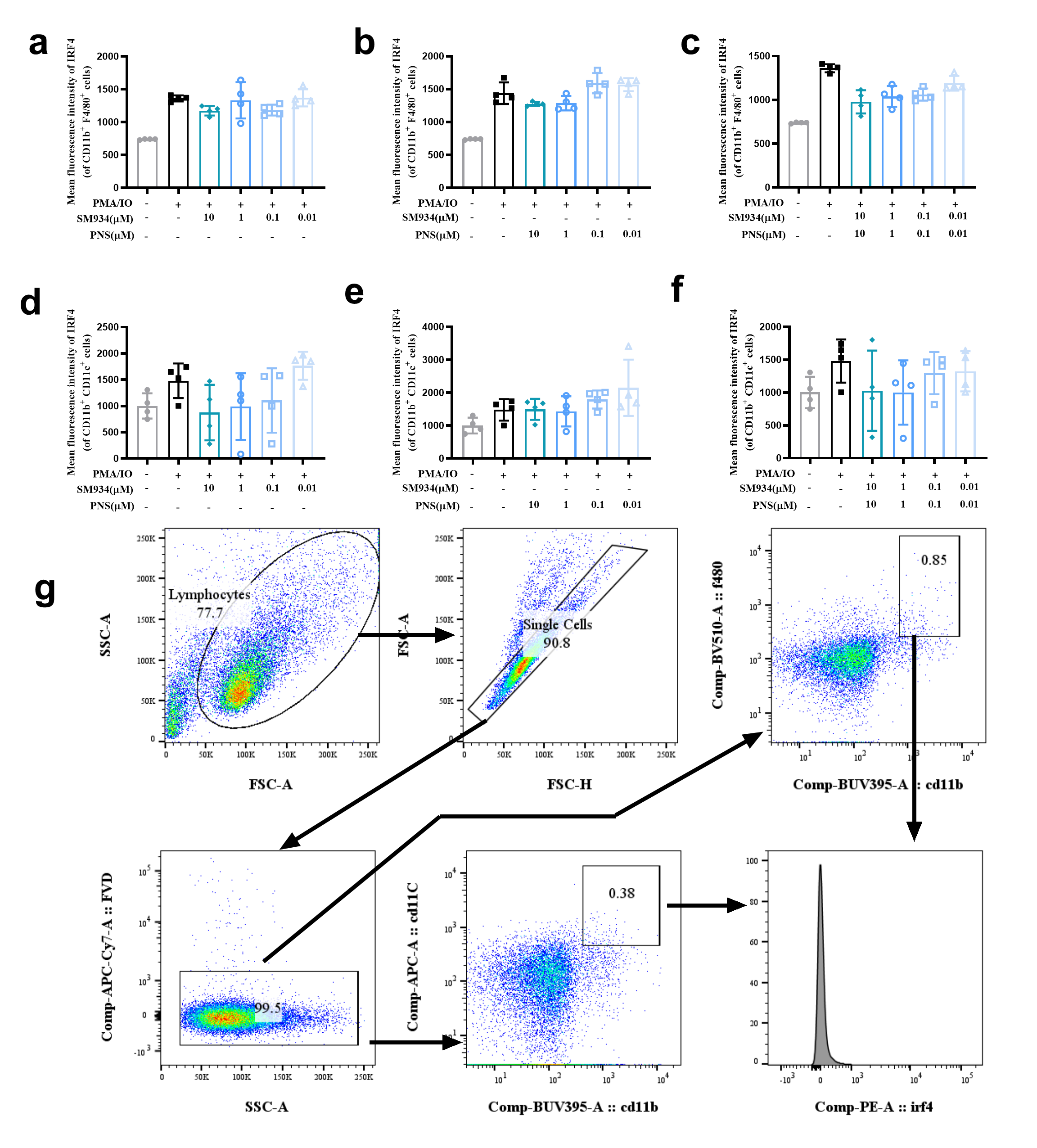


**Fig. S14. SM934 or PNS treatment hardly affect IRF4 expression in macrophages or dendritic cells.** Expression of IRF4 on CD11b^+^F4/80^+^ cells (a-c) and CD11b^+^CD11c^+^ cells (d-f) from the spleens of MRL/*lpr* mice. Cells were treated with the indicated compounds for 24 hours in the presence of 1 µg/mL PMA and 1 µM IO at 37°C and 5% CO_2_. (c) Gating strategy for the detection of IRF4 on macrophage (CD11b^+^F4/80^+^) and dendritic cells (CD11b^+^CD11c^+^) cells. n=4 per group. Statistic data were represented as mean ± SD.

Figure. S15.


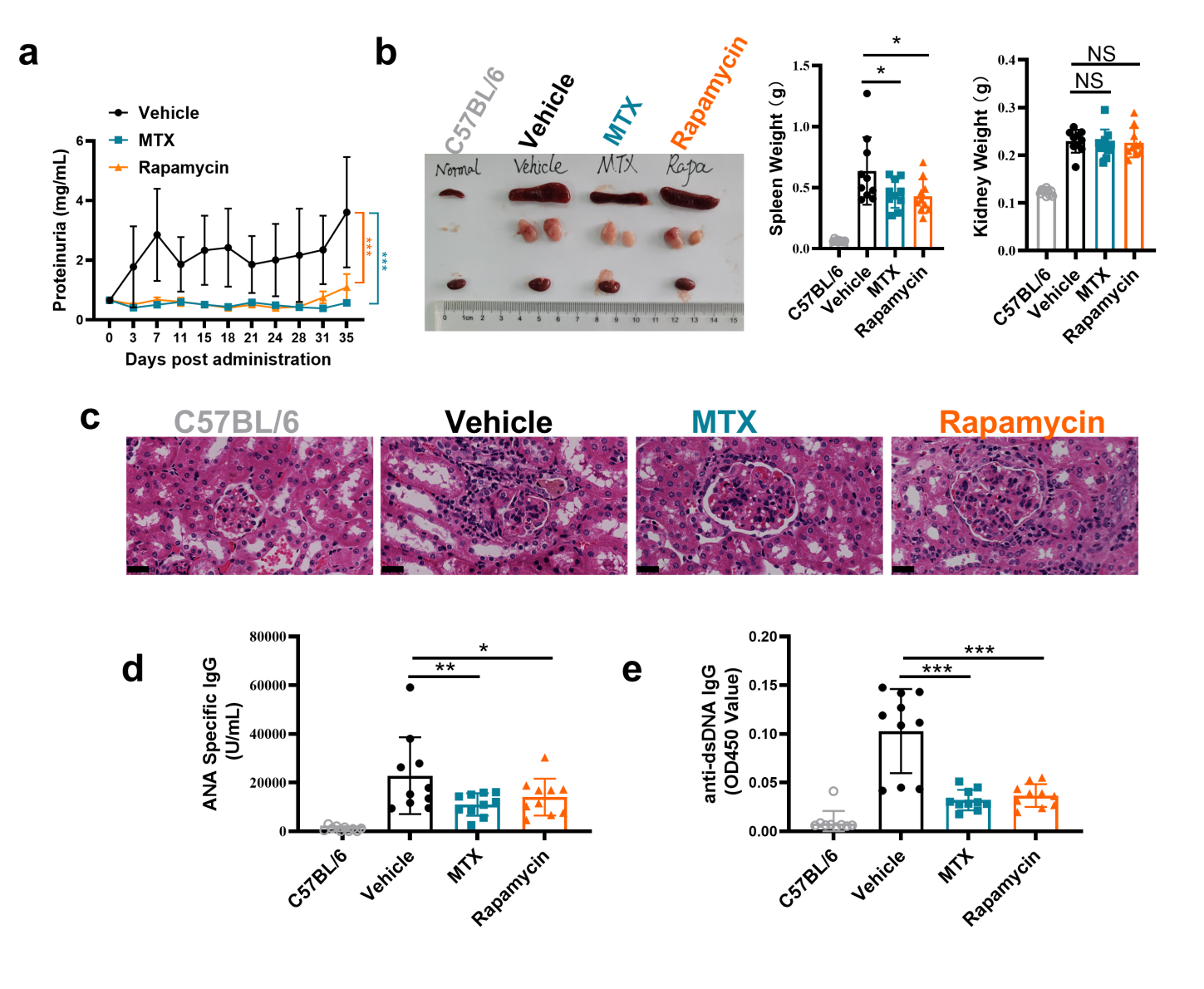


**Fig. S15. Treatment with MTX and rapamycin attenuates lupus-like symptoms and suppressed IRF4 expression in MRL/*lpr* mice.** MRL/*lpr* mice were treated with MTX (1 mg/ kg) or Rapamycin (1 mg/kg) for 5 weeks (n=10 per group). (a) The proteinuria level was monitored once a week. (b) The spleens, lymph nodes, and kidneys of the MRL/*lpr* mice in each group were photographed (left panel) and spleen weight and kidney weight were measured at the end of the treatment. (c) Photomicrographic representation of pathological changes of kidney glomeruli was detected by H&E (scale bars: 100 μm). (d, e) Levels of serum ANA-specific IgG, anti-dsDNA autoantibodies were detected by ELISA. Statistic data were represented as mean ± SD. * P<0.05, ** P<0.01, *** P<0.001 versus the vehicle group.

Figure. S16.


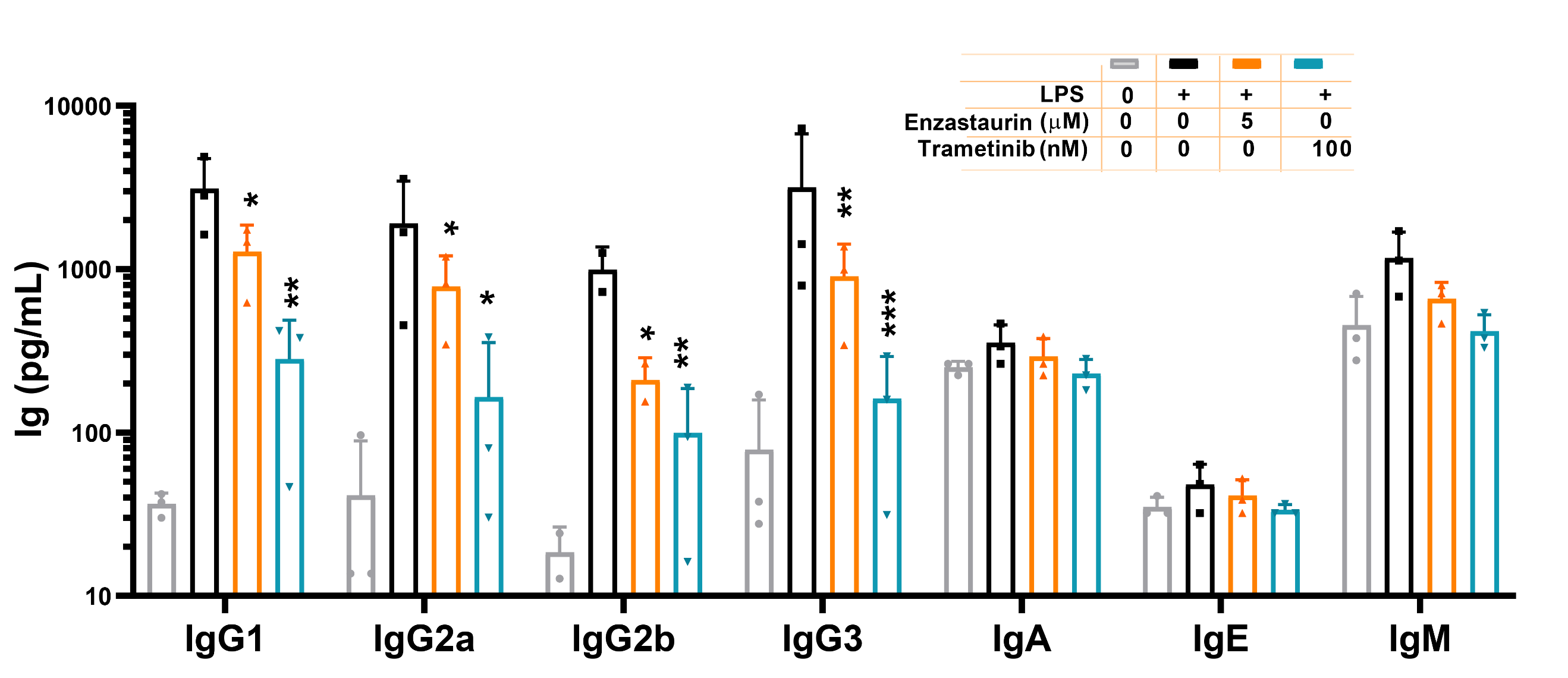


**Fig. S16. Enzastaurin and Trametinib inhibited IgGs production from LPS-stimulated CD19^+^ B cells.** CD19^+^ B cell from C57BL/6 mice were stimulated with/without LPS (10 μg/mL) for 120 h in the presence or absence of Enzastaurin (5 μM) or Trametinib (100 nM). Levels of antibody in culture supernatants were determined. n=3 per group. Statistic data were represented as mean ± SD. * P<0.05, ** P<0.01, *** P<0.001 versus the LPS group.

Figure. S17.

**
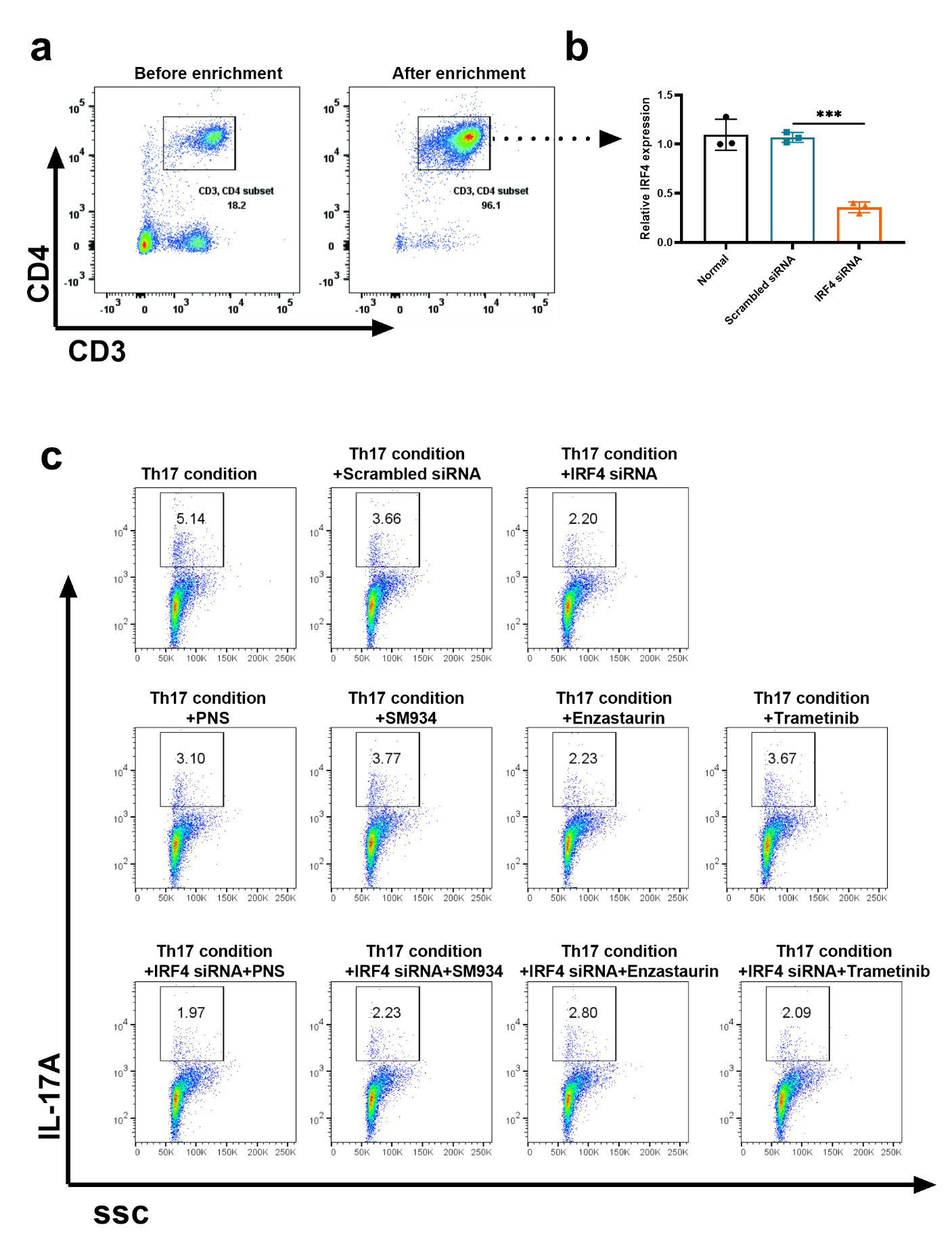
**

**Fig. S17. Percentage of IL-17^+^ cells in CD3^+^CD4^+^ T cells under Th17-polarizing condition.**

(a, b) Interference efficiency of IRF4 siRNA. (c) *IRF4* intact or si*IRF4* naïve CD4^+^ T cells were treated with PNS (10 μM), SM934 (10 μM), Enzastaurin (5 μM) or Trametinib (100 nM) cultured in Th17-polarizing conditions. Representative plots were shown. n=3 per group. Statistic data were represented as mean ± SD. *** P<0.001,
