# Supplementary material for: Repression of interferon regulatory factor-4 (IRF4) hyperactivation restricts murine lupus
Source: Signal Transduct Target Ther. 2023 May 22;8:188. doi: 10.1038/s41392-023-01413-8 (PMC10200790; doi:10.1038/s41392-023-01413-8)

Supplementary Materials for

Repression of Interferon Regulatory Factor-4 (IRF4) Hyperactivation Restricts Murine Lupus

Shijun He^1, 2 #,*^, Huihua Ding^3, #^, Li Chen^2#^, Yiwei Shen ^3^, Yuting Liu^2^, Fenghua Zhu^2^, Xiaoqian Yang^2^, Nan Shen^3^, Zemin Lin^2 #,*^, Jianping Zuo^2*^

Correspondence to: Shijun He (heshijun@shutcm.edu.cn, heshijun@simm.ac.cn); Zemin Lin (linzemin@simm.ac.cn); Jianping Zuo (jpzuo@simm.ac.cn)

^1^ Innovation Research Institute of Traditional Chinese Medicine, Shanghai University of Traditional Chinese Medicine, Shanghai, China;

^2^ Laboratory of Immunopharmacology, State Key Laboratory of Drug Research, Shanghai Institute of Materia Medica, Chinese Academy of Sciences, Shanghai, China;

^3^ Department of Rheumatology, Ren Ji Hospital, School of Medicine, Shanghai Jiao Tong University, Shanghai, China.

**This PDF file includes:**

Original and uncropped films of Western blots

Supplementary Fig. S9d


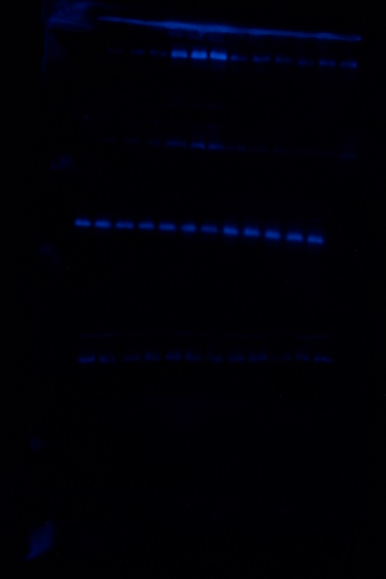

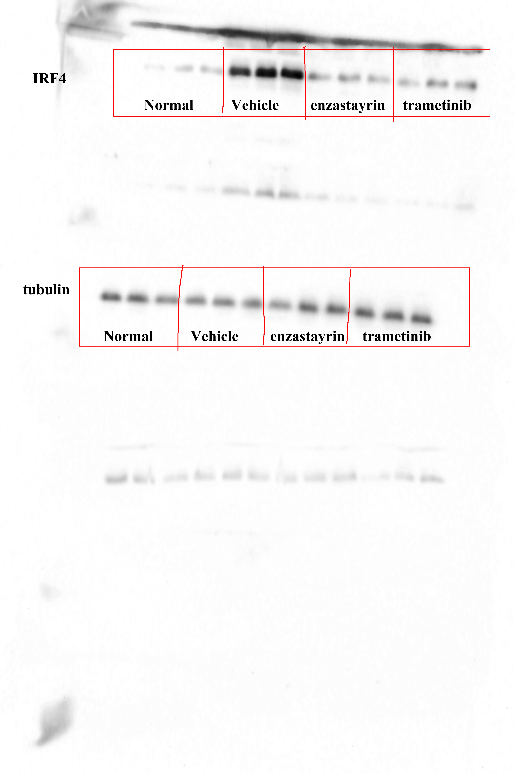

Supplement: Supplementary file 2 — Original and uncropped films of Western blots [file 41392_2023_1413_MOESM2_ESM.docx]
